# Climate change and broiler production

**DOI:** 10.1002/vms3.1416

**Published:** 2024-03-20

**Authors:** Oyegunle Emmanuel Oke, Oluwaseun Ayomide Akosile, Victoria Anthony Uyanga, Folasade Olukemi Oke, Aderanti Ifeoluwa Oni, Kokou Tona, Okanlawon Mohammed Onagbesan

**Affiliations:** ^1^ Department of Animal Physiology Federal University of Agriculture Abeokuta Nigeria; ^2^ Centre of Excellence in Poultry Sciences University of Lome Lome Togo; ^3^ Department of Animal Science Iowa State University Ames Iowa USA; ^4^ Department of Agricultural Economics and Farm Management Federal University of Agriculture Abeokuta Nigeria

**Keywords:** adaptation, broiler, climate change, environments, tropics

## Abstract

Climate change has emerged as a significant occurrence that adversely affects broiler production, especially in tropical climates. Broiler chickens, bred for rapid growth and high meat production, rely heavily on optimal environmental conditions to achieve their genetic potential. However, climate change disrupts these conditions and poses numerous challenges for broiler production. One of the primary impacts of climate change on broiler production is the decreased ability of birds to attain their genetic potential for faster growth. Broilers are bred to possess specific genetic traits that enable them to grow rapidly and efficiently convert feed into meat. However, in tropical climates affected by climate change, the consequent rise in daily temperatures, increased humidity and altered precipitation patterns create an unfavourable environment for broilers. These conditions impede their growth and development, preventing them from reaching their maximum genetic influence, which is crucial for achieving desirable production outcomes. Furthermore, climate change exacerbates the existing challenges faced by broiler production systems. Higher feed costs impact the industry's economic viability and limit the availability of quality nutrition for the birds, further hampering their growth potential. In addition to feed scarcity, climate change also predisposes broiler chickens to thermal stress. This review collates existing information on climate change and its impact on broiler production, including nutrition, immune function, health and disease susceptibility. It also summarizes the challenges of broiler production under hot and humid climate conditions with different approaches to ameliorating the effects of harsh climatic conditions in poultry.

## Graphical abstract

In this review, the latest data on climate change's effects on broiler production – including those on health, immunity and disease susceptibility – are compiled. The difficulties of producing broilers in hot, humid climates are also summarized, along with several methods for reducing the negative impacts of hard weather on poultry.

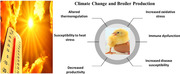

## 1 INTRODUCTION

Globally, agricultural systems have been negatively impacted by climate change‐related factors (Arora, 2019). This is often characterized by global warming and changes in weather patterns (Ajayi et al., 2022; Dube et al., 2016). According to Sundström et al. (2014), escalating temperatures, erratic rainfall, protracted droughts and an increase in the frequency of extreme weather events pose significant challenges to food production and security. These changes disrupt the delicate balance of the ecosystems, affecting the growth, development and productivity of crops and livestock. The tropics are particularly susceptible to the effects of climate change due to their high temperatures and diverse ecosystems (Oke et al., 2022; Thomson et al., 2015). This review critically examines the challenges and opportunities bedevilling broiler chicken production as it embraces the ongoing onslaught of climate change and its antecedent of global warming, especially in the tropical regions of the world.

The changing climate poses significant challenges to achieving optimal broiler growth and production outcomes. It disrupts the delicate balance of environmental factors necessary for the expression of broilers’ genetic potential. Consequently, understanding and mitigating the effects of climate change on broiler production is crucial for ensuring food security and sustainability in the tropics. In this review, the specific implications of climate change on broiler production in tropical climates are discussed. Moreover, the genetic potential and growth limitations of broilers under changing climatic conditions, the scarcity and cost of poultry feed driven by climate‐related disruptions, the challenges posed by thermal stress and heat management, and the impacts on broiler immune functions and disease susceptibility are explored. Furthermore, the adaptation strategies and potential policy implications for promoting sustainable broiler production in the face of climate change are presented. By examining the interplay between climate change and broiler production in the tropics, this review aims to shed light on the challenges faced by the industry and explore potential avenues for resilience and sustainable practices. Ultimately, understanding these dynamics is essential for addressing the impacts of climate change and ensuring the long‐term viability of broiler production in the tropics.

## 2 TRENDS AND CHANGES IN BROILER MEAT PRODUCTION

**FIGURE 1:**
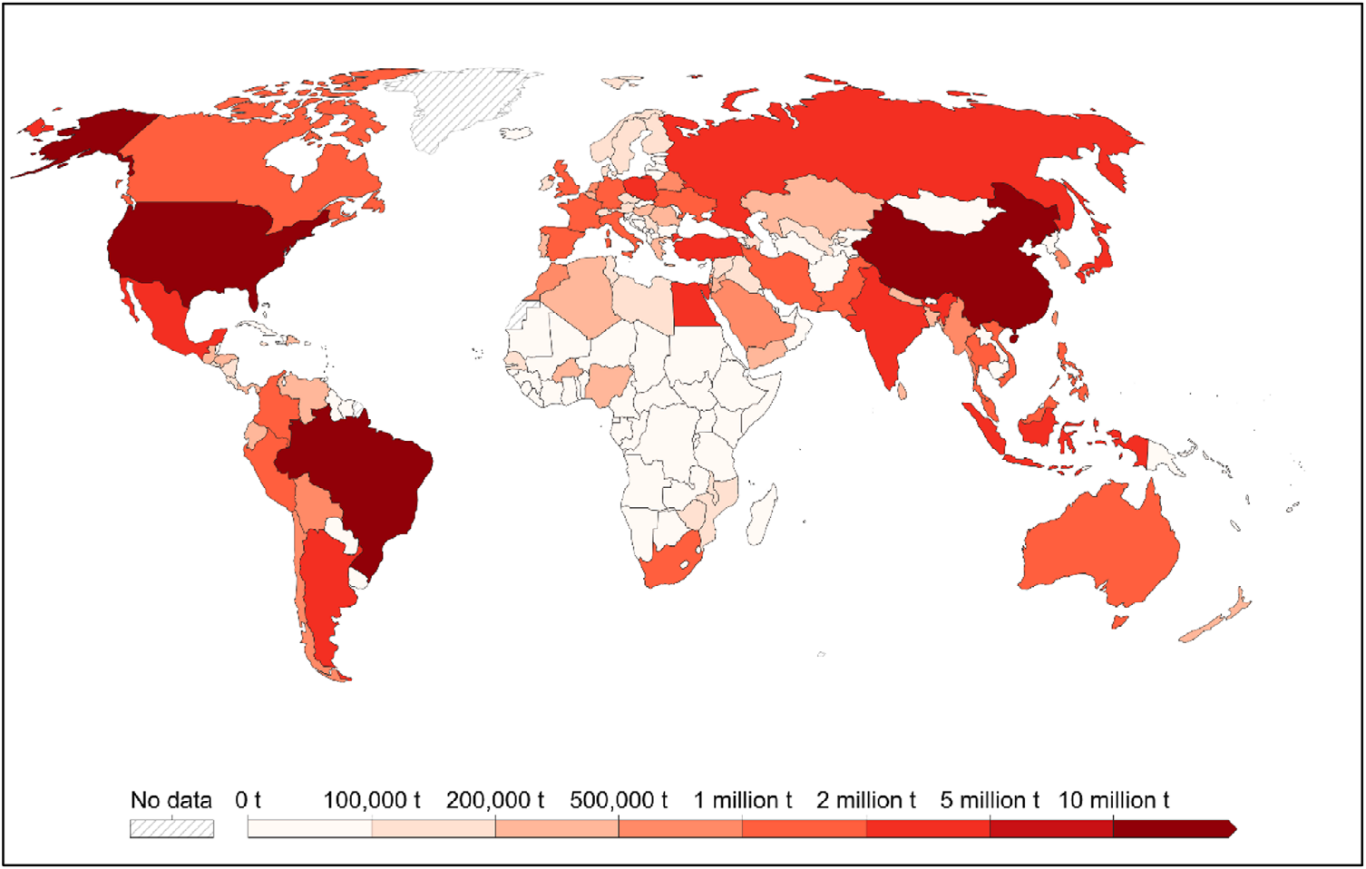
Global chicken meat production in 2021 (measured in tonnes per year). *Source*: Food and Agriculture Organization of the United Nations.

Broiler production refers to the rearing of fast‐growing meat‐type chickens specifically bred for meat production and plays a vital role in the global food supply (Hinsemu et al., 2018). In the tropics, broiler production is of particular importance due to the high demand for poultry meat and the suitability of the climate for year‐round production (Mottet & Tempio, 2017). Chicken meat production plays a substantial role in meeting global protein supply due to its increased acceptability, short production cycle and the rapid growth of the poultry industry in recent decades. According to the Food and Agriculture Organization of the United Nations, chicken meat production has increased significantly over the last decades across the globe (Figure 1).

The top five producing countries in 2021 were the United States, China, Brazil, Russia and Indonesia. The world's production of broiler meat production rose to about 121.5 million metric tons, with a relative increase of 107% between 2000 and 2021. Similarly, world regions, including Asia, Europe, North America, South America, Oceania and Africa, had witnessed a profound increment in chicken meat production with a relative change of 132%, 109%, 55%, 140%, 113% and 161% from 2000 to 2021, respectively. Africa had the highest relative change, having a rapidly growing poultry industry with greater potential for development. The different African regions, including Eastern Africa, Middle Africa, Northern Africa, Southern Africa and Western Africa, had a relative change of about 169%, 171%, 190%, 132% and 116% in their chicken meat production from 2000 to 2021, respectively. Importantly, low‐income countries, lower middle‐income countries, upper middle‐income countries, Least Developed Countries and low‐income food‐deficit countries contributed significantly to chicken meat production with a higher relative change compared to the high‐income countries (Table 1). The rapid growth in broiler production in these growing economies may be related to their rapidly increasing population growth, industrialization and urbanization, investment to meet protein supply, intensive and vertically integrated production systems, commercialization of poultry industries into large enterprises and the improved genetic potential of modern‐day broilers.

**TABLE 1:** Chicken meat production trend across global regions between 2000 and 2021.

Countries/Global region	2000	2021	Absolute change	Relative change in %
World	58,698,450	121,588,360	62,889,910	107
Asia	18,615,080	43,100,190	24,485,110	132
Europe	9,317,664	19,450,668	10,133,004	109
North America	17,777,672	27,522,254	9,744,582	55
South America	9,453,123	22,653,408	13,200,285	140
Oceania	731,286	1,555,450	824,165	113
Africa	2,777,624	7,250,987	4,473,363	161
Eastern Africa (FAO)	326,906	878,492	551,586	169
Middle Africa (FAO)	59,383	160,877	101,494	171
Northern Africa (FAO)	1,164,852	3,372,508	2,207,656	190
Southern Africa (FAO)	834,402	1,936,201	1,101,799	132
Western Africa (FAO)	418,081	902,910	484,829	116
High‐income countries	27,261,688	42,418,148	15,156,460	56
Low‐income countries	606,558	1,333,952	727,394	120
Lower middle‐income countries	6,128,900	22,273,040	16,144,141	263
Upper middle‐income countries	23,992,490	55,067,932	31,075,442	130
Least Developed Countries (FAO)	961,320	2,654,462	1,693,142	176
Low‐income food‐deficit countries (FAO)	2,145,720	7,966,226	5,820,506	271

*Notes*: FAO refers to country/regional categorization based on the framework of the Food and Agriculture Organization of the United Nations.

*Source*: Food and Agriculture Organization of the United Nations. OurWorldInData.org/meat‐production.

The tropics offer favourable conditions for broiler farming, such as abundant natural resources, favourable disease profiles and accessibility to global markets (Saeed et al., 2019). Therefore, the impact of climate change on broiler production in tropical regions must be carefully addressed, especially with the evolving nature and new understanding arising from studies of climate change and its impacts on life forms.

## 3 CARBON FOOTPRINT FROM BROILER PRODUCTION

According to Müller et al. (2020), the total amount of greenhouse gases (GHG; comprising carbon dioxide [CO_2_], methane [CH_4_] and nitrous oxide [N_2_O]) created by anthropogenic activity is known as the ‘carbon footprint’. The carbon footprint serves as a measurement of the GHG emissions generated from a certain activity, sector or enterprise (Garnett, 2011). Despite having a relatively lesser carbon footprint than other animal production systems, the broiler industry nonetheless contributes significantly to GHG emissions (Leinonen & Kyriazakis, 2016). Several processes undertaken during broiler chicken production contribute directly and/or indirectly to GHG emissions. Along with direct energy use on farms, post‐farm meat processing and shipping, and manure storage and processing and feed production account for the majority (78%) of GHG emissions in the broiler industry (Herrero et al., 2016). As such, the efficiency and effectiveness through which broilers convert feed into meat are crucial determinants of the quantity and intensity of GHG emissions resulting from broiler production.

Essentially, the three major sources of GHG emissions from broiler production are manure management, energy use and feed production. The carbon footprint of broiler production is influenced by feed production, which includes land use change, fertilizer application and transportation (Nijdam et al., 2012). Emissions are further increased by the use of energy for heating, ventilation and lighting, which includes fossil fuels and electricity. Last but not least, methane, a powerful GHG, is released due to manure management, including storage and disposal (Borhan et al., 2012). Carbon dioxide, methane and nitrous oxide have been identified as the main sources of GHG emissions in relation to farm operations (on‐farm activities). The source of the majority of the nitrous oxide emissions in the industrial, modern broiler farm was bedding (Dunkley et al., 2015).

**FIGURE 2:**
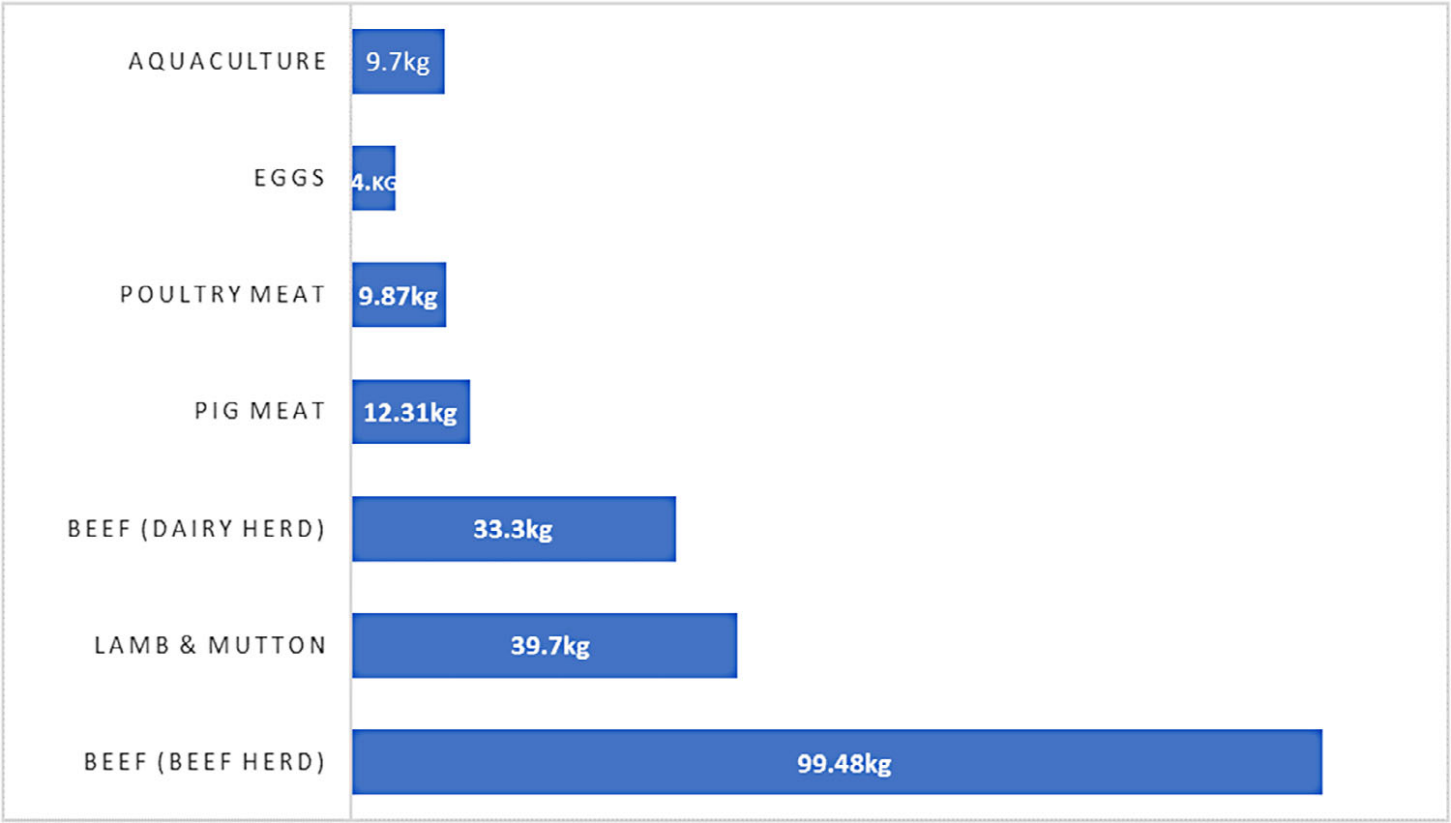
Greenhouse gas emissions per kilogramme of food product (kgCO₂eq per kilogramme). Emissions were measured in carbon dioxide equivalents. ***Source***: Adapted from OurWorldInData.org/environmental‐impacts‐of‐food (Poore & Nemecek, 2018).

A recent study exploring the environmental impacts of food products (Figure 2) uncovered that poultry meat contributed 9.87 kg CO₂eq per kilogramme, which was lower than that obtainable from cattle (beef and dairy herd), lamb and mutton and pig meat production (Poore & Nemecek, 2018). According to estimates, chickens will produce 0.6 Gt of CO_2_‐equivalent emissions or contribute to 8% of total emissions from the animal industry (Eisen & Brown, 2021). Specifically, for broiler production, 78% of feed production, 8% of direct energy consumption on farms, 7% of post‐farm meat processing and shipping and 6% of manure storage and processing are responsible for GHG emissions (MacLeod et al., 2019).

The carbon footprint from broiler production is influenced by several factors. Due to the fact that the manufacture and distribution of feed ingredients increase emissions, the content and source of the feed are very important. By lowering the amount of feed required per unit of meat produced, effective feed conversion ratios and good nutrient utilization can help minimize the carbon footprint. Utilizing renewable energy sources and improving the energy efficiency of broiler housing can dramatically reduce emissions. Furthermore, methane can be captured and used for sustainable manure management techniques like anaerobic digestion or composting. Therefore, the environmental impact of broiler production must be addressed, even though it has a relatively smaller carbon footprint than other livestock production systems. The carbon footprint of broiler production can be greatly decreased by putting plans into place to optimize feed, increase energy efficiency, adopt sustainable manure management practices and use genetic selection. Promoting sustainable practices that reduce GHG emissions that can ensure an environmentally friendly broiler production system requires further study, innovation and cooperation among the various stakeholders in the poultry sector.

## 4 CLIMATE CHANGE AND BROILER PRODUCTION

**FIGURE 3:**
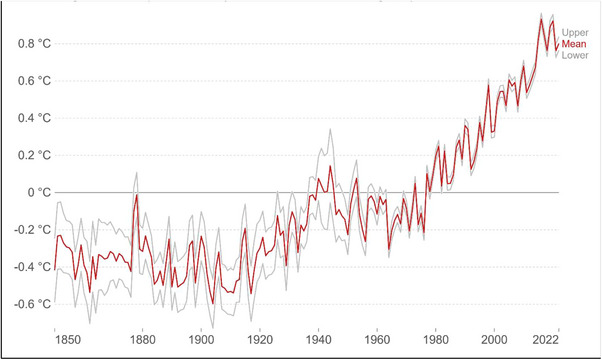
The anomaly in the global average of land–sea temperature relative to the average of the period between 1961 and 1990. The red line depicts the average annual temperature trend over time, whereas the grey lines depict the upper and lower boundaries of the 95% confidence intervals. ***Source***: Met Office Hadley Centre (HadCRUT5) OurWorldInData.org/co2‐and‐greenhouse‐gas‐emissions.

Climate change and its associated global warming have become increasingly prominent worldwide (IFAD, 2009). This is primarily driven by anthropogenic emissions of carbon dioxide and other GHGs. Global temperatures have risen with an increase of ∼1–1.2°C in present times relative to the ‘pre‐industrial times’ (Intergovernmental Panel on Climate Change [IPCC], 2014). Over the last decades, there have been year‐to‐year fluctuations in temperature and a sharp increase of approximately 0.7°C compared to the 1961–1990 baseline (Figure 3). These changes in climatic conditions have potential consequences on the physical, ecological and health impacts of living organisms, including the incidence of extreme weather events, such as floods, droughts, and heatwaves (IPCC, 2018).

The impact of climate change is increasingly evident in the livestock subsector of the economy (Baumgard et al., 2012). One of the primary concerns is the rise in global temperatures and the subsequent increase in environmental temperature. Although the livestock subsector has been recognized for contributing 18% of global GHG emissions (Bellarby et al., 2013), poultry production is known to leave a comparatively smaller carbon footprint per unit of product compared to ruminant production (Chhabra et al., 2009; Lassey, 2007). However, several studies have asserted that poultry species are more susceptible to the adverse effects of elevated temperatures than other food animal species (Oke et al., 2021a; Uyanga et al., 2023). The poultry industry has undergone significant development (Daghir, 1995) to meet the food demands of the ever‐growing populations (Rehman et al., 2017). However, this progress may be compromised due to the increasing incidence of heat stress in tropical regions (Renaudeau et al., 2012; Uyanga et al., 2022). Therefore, it is imperative to explore, discover and conserve all available biodiversity and genetic resources within our animal heritage to enable adaptation to these changing climatic conditions.

### 4.1 Impacts of climate change on broiler production in the tropics

Broiler production in tropical regions possesses unique characteristics and faces specific challenges due to the distinct environmental conditions prevalent in these areas (Pius et al., 2021). Tropical climates are characterized by high temperatures, high humidity and often a distinct wet and dry season pattern (Tamerius et al., 2013). These conditions create both advantages and challenges for broiler production. One advantage is that the relatively warm temperatures throughout the year provide favourable conditions for year‐round production without the need for extensive heating systems. Therefore, understanding these factors is essential for comprehending the impact of climate change on broiler production in the tropics.

#### 4.1.1 Altered thermoregulation

One of the primary effects of climate change on broiler production is the increasing ambient temperatures in many regions. Temperature regulation is particularly important for broilers, as their metabolism generates heat, and they rely on their surroundings to dissipate it (Renaudeau et al., 2012). In tropical climates, where temperatures can be high and heat stress is common, maintaining an optimal thermal environment becomes challenging (Sherwood & Huber, 2010). Thermal stress occurs when broilers are exposed to temperatures above their thermoneutral zone, which is the range of temperatures where birds can maintain their body temperature without expending extra energy (Gous & Morris, 2005). All living organisms have a thermal comfort range, which is a range of surrounding environmental temperatures (Djongyang et al., 2010). In poultry farms, heat stress sets in when the ambient temperature exceeds 25°C, and the cold temperature is a temperature less than 20°C on average; the optimum is located mostly between 22 and 25°C (Abu‐Dieyeh, 2006). High relative humidity could modify animals’ perception of the heat (Yahav et al., 2000). Then, the temperature must be associated with relative humidity in order to properly assess heat stress. These temperatures are optimal for biological functions. Elevated temperatures and humidity pose significant challenges to broiler production, leading to thermal stress and impacting various aspects of broiler performance (Bilal et al., 2021). The neuroendocrine system is also impaired as a result of high elevated temperature. The hypothalamic–pituitary–adrenal axis becomes activated in chickens exposed to high ambient temperatures, and higher plasma corticosterone concentrations result in decreased systemic levels and functioning (Binsiya et al., 2017; Lo Sauro et al., 2008).

Understanding the effects of thermal stress and implementing effective heat management strategies are crucial for maintaining optimal broiler health and productivity (Ranjan et al., 2019). It is generally accepted that broilers above 2 weeks of age will grow optimally at a temperature range of 20–24°C (Yani et al., 2014). Broilers are highly sensitive to heat stress, as their natural thermoregulatory mechanisms are easily overwhelmed in high‐temperature conditions (Nawaz et al., 2021). Moreover, thermal stress affects nutrient utilization in broilers. The body's response to heat stress diverts energy away from growth‐related processes, including nutrient assimilation and utilization. This results in reduced nutrient absorption and metabolic inefficiencies, further hampering broiler growth potential.

#### 4.1.2 Susceptibility to heat stress

Poultry species, including broilers, are highly sensitive to heat stress, which occurs when the environmental temperature exceeds their thermoneutral zone (Akosile, Sogunle et al., 2023; Akosile, Kehinde et al., 2023; Kpomasse et al., 2021; Kpomasse, kouame et al., 2023; Pawar et al., 2016). Heat stress negatively affects livestock growth, feed intake, nutrient utilization and immune functions (Abbas et al., 2022; Kpomasse, Oso et al., 2023; Yahav, 2004). These adverse effects result in reduced production efficiency, decreased meat yield and increased susceptibility to diseases (Song & King, 2015).

In the latest work by Mohammed et al. (2018), broilers subjected to heat stress reduced feed intake and higher feed intake at day 42. Other studies also show lower feed intake and altered performance as a result of high thermal temperature induced high ambient temperature (Al‐Fataftah et al., 2014; Calik et al., 2022; Rosa et al., 2007; Nanto‐Hara et al., 2020). High ambient temperatures are accompanied by changes in the physiology of broiler birds in order to regulate their internal environment, thereby leading to a decrease in internal temperature (Soliman & Safwat, 2020). Broiler birds show different behaviours to thermal stress, either by panting or less time feeding or drinking, or wing flapping with less time walking and high resting time, as shown by Mohammed et al. (2018).

Broilers are genetically selected for their ability to efficiently convert feed into meat within a short span of time (Willems et al., 2013). To achieve their genetic potential, broilers require an environment that supports their physiological needs, which includes adequate ventilation, appropriate temperature and humidity levels, access to clean water and proper sanitation (Haque et al., 2020).

#### 4.1.3 Decreased productivity

Failure to provide suitable ventilation and cooling systems can result in reduced feed intake, slower growth rates and increased susceptibility to diseases (Soliman & Safwat, 2020). Heat stress affects broiler feed intake, as birds reduce feed consumption to cope with high temperatures (Gonzalez‐Esquerra & Leeson, 2006). Reduced nutrient intake leads to inadequate essential nutrients required for optimal immune function (Maggini et al., 2018).

#### 4.1.4 Water unavailability

The availability and quality of water play a crucial role in broiler production. Clean and readily accessible water sources are necessary for hydration, digestion and overall well‐being of poultry (Abioja & Abiona, 2021; El Sabry et al., 2023; Aggrey et al., 2023). In tropical regions, where rainfall patterns may be unpredictable or concentrated within a specific season, ensuring consistent access to water can be challenging. Climate change further exacerbates this challenge by altering precipitation patterns, potentially leading to water scarcity or excessive flooding, which can disrupt broiler production.

Climate change also disrupts precipitation patterns, resulting in unpredictable and uneven rainfall distribution. This poses challenges for broiler production as it affects the availability and quality of water resources, which are essential for the birds’ hydration, digestion and overall well‐being. Water scarcity or excessive flooding can occur, both of which negatively impact broiler growth and health. Insufficient water availability can lead to dehydration, reduced feed intake and impaired nutrient absorption. Reduction in water intake with a subsequent increase in pulse and respiratory rates and panting further impacts broiler performance and overall productivity (Bruno et al., 2011; Lara & Rostagno, 2013; Oloyo, 2018), reduces reproductive performance (Das et al., 2016) and alters respiratory functions (Polsky & von Keyserlingk, 2017).

#### 4.1.5 Extreme weather events

Extreme weather events caused by climate change pose a serious threat to livestock production. Climate change is associated with an increase in extreme weather events, such as storms, hurricanes and prolonged droughts (Diez et al., 2012). These events constitute a significant risk to broiler production systems. Climate change may influence seasonal patterns, resource availability variations and crop productivity, all of which may be affected, resulting in a reduction in the quantity and quality of feedstuffs available for livestock production (Konapala et al., 2020). The adverse impact of extreme weather events on grain yield has been documented (Lobell et al., 2011). Moreover, chickens will experience more heat stress as heatwave frequency and duration rise (Vitali et al., 2015). Additionally, severe storms can damage poultry housing infrastructure, disrupt power supply and compromise biosecurity measures.

#### 4.1.6 Feed availability

The abundance of natural resources, such as water and vegetation, can facilitate feed production and availability. Climate‐related disruptions, such as droughts and floods, can significantly impact the production and availability of key feed ingredients, such as grains and soya beans. This scarcity leads to a rise in feed prices, directly impacting the profitability and sustainability of broiler production. Higher feed costs not only affect the economic viability of the industry but also limit the availability of quality nutrition for the birds, further compromising their growth potential.

Prolonged droughts lead to feed scarcity, increased feed costs and reduced access to quality nutrition for broilers, further hindering their growth and development. In addition to these direct impacts, climate change indirectly affects broiler production through its influence on the availability and cost of feed ingredients (Abioja & Abiona, 2021).

Broilers tend to eat less due to the physiological effects of heat stress. This decreased feed intake directly affects nutrient availability for growth and development, leading to slower growth rates and compromised feed conversion ratio.

#### 4.1.7 Immune dysfunction

Climate change poses multifaceted challenges to broiler production, impacting various aspects of the birds’ physiology and health (Roberts et al., 2015). One critical aspect is the effect of climate change on broiler immune functions, which can lead to compromised immunity and increased susceptibility to infectious diseases (Wickramasuriya et al., 2022). Understanding these implications is vital for implementing strategies to safeguard broiler health and ensure sustainable production amid changing climatic conditions.

Elevated temperatures and heat stress associated with climate change have direct effects on broiler immune functions (Soliman & Safwat, 2020). Heat stress triggers a series of physiological responses that divert energy away from immune system functions, impairing its ability to combat pathogens effectively (Moberg, 2000). Heat stress induces the release of stress hormones, such as corticosterone, which suppress the immune response and compromise the production of immune cells (Nardocci et al., 2014). Additionally, high temperatures can impact the development and function of lymphoid tissues responsible for immune cell maturation and activation (Hirakawa et al., 2020). Reduced lymphoid tissue function leads to decreased antibody production and an impaired immune system, making broilers more susceptible to infectious agents (Calder & Kew, 2002).

#### 4.1.8 Increased disease susceptibility

The compromised immune systems of birds under heat stress render them more susceptible to infectious diseases (Hamouda et al., 2023; Mehaisen et al., 2016; Quinteiro‐Filho et al., 2012). Pathogens that might have been effectively controlled under optimal temperature conditions can now proliferate in broiler flocks, leading to disease outbreaks (Nørrung et al., 2009). Bacterial, viral and parasitic infections can become more prevalent, affecting the overall health and productivity of the birds (Nørrung et al., 2009). Furthermore, broilers with compromised immune systems may experience prolonged recovery times from infections, leading to reduced growth rates and increased mortality rates (Umar et al., 2017). Heat stress directly affects immune system responses, hindering the birds’ ability to combat pathogens effectively. The implications of compromised immune systems on disease susceptibility can result in disease outbreaks, increased mortality and reduced growth rates, leading to economic losses in broiler production (Swaggerty et al., 2019).

**FIGURE 4:**
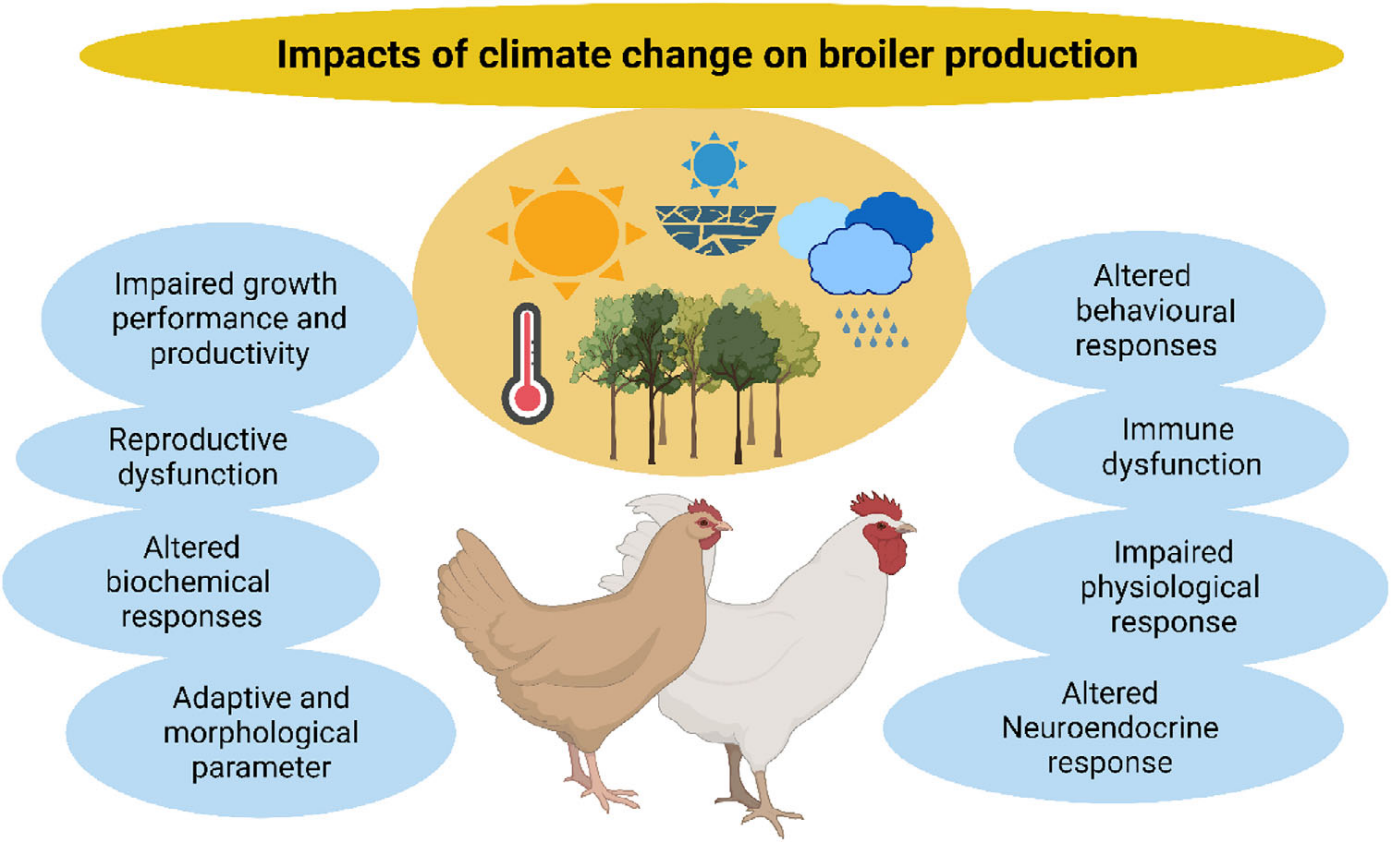
Diagram illustrating the impact of climate change on poultry production.

Climate change has significant implications for broiler production, as illustrated in Figure 4, with specific effects that directly impact the industry's productivity and sustainability. The link between reduced nutrient intake, infectious diseases and broiler health further exacerbates the vulnerability of birds to climate‐induced stressors (Soumya et al., 2022). To address these challenges, broiler producers must adopt heat management strategies, optimize nutrition and implement disease prevention and control measures. By prioritizing broiler health and immunity, the industry can enhance resilience and ensure sustainable production in the face of climate change. Additionally, ongoing research and advancements in breeding and management practices are essential for developing heat‐tolerant broiler strains and implementing adaptive measures to maintain broiler health and performance in a changing climate.

## 5 ADAPTATION STRATEGIES FOR SUSTAINABLE BROILER PRODUCTION IN THE TROPICS

As climate change continues to impact broiler production, it is crucial to implement adaptation strategies to ensure the industry's sustainability and resilience. These strategies aim to mitigate the effects of climate change on broiler health, performance and overall productivity. This review provides an overview of adaptation measures, with a focus on improved housing systems, ventilation, cooling mechanisms, alternative feed options and other sustainable practices for broiler production.

Adaptation measures in broiler production involve implementing practices that enhance the birds’ ability to cope with changing climatic conditions (Renaudeau et al., 2012). These measures aim to create a comfortable and controlled environment for broilers, reduce heat stress, optimize nutrition and improve overall welfare. Effective adaptation strategies help minimize the negative impacts of climate change on broiler growth and performance, ensuring sustainable production and economic viability (Vandana et al., 2021).

### 5.1 Environmental strategies

Upgrading housing systems is a key adaptation measure to address the challenges of climate change (Shaw et al., 2010). Modern broiler housing should be designed to maximize ventilation, airflow and temperature control. Ventilation and air movement are crucial aspects of heat management in broiler production systems, particularly in tropical climates where high temperatures and humidity levels prevail (Nienaber & Hahn, 2007). Proper ventilation is essential for removing excess heat, moisture and airborne pollutants from the broiler house, creating a more comfortable environment for the birds (Bucklin et al., 2009).

Adequate air movement is necessary to ensure the removal of stale air and the circulation of fresh air throughout the broiler house. Stale air can contain a build‐up of heat, humidity and harmful gases such as ammonia and carbon dioxide, which can have detrimental effects on broiler health and performance (Butcher & Miles, 2012). Effective ventilation systems should be designed to facilitate the exchange of stale air with fresh air from outside, promoting better air quality and reducing the heat load within the facility (Chua et al., 2013). Furthermore, ventilation and air movement contribute to managing humidity levels within the broiler house. High humidity can exacerbate the heat load on the birds and create an environment favourable for the growth of pathogens and the occurrence of respiratory diseases. By effectively removing excess moisture from the house, ventilation systems help to reduce humidity levels and maintain a drier and healthier environment for the broilers.

**FIGURE 5:**
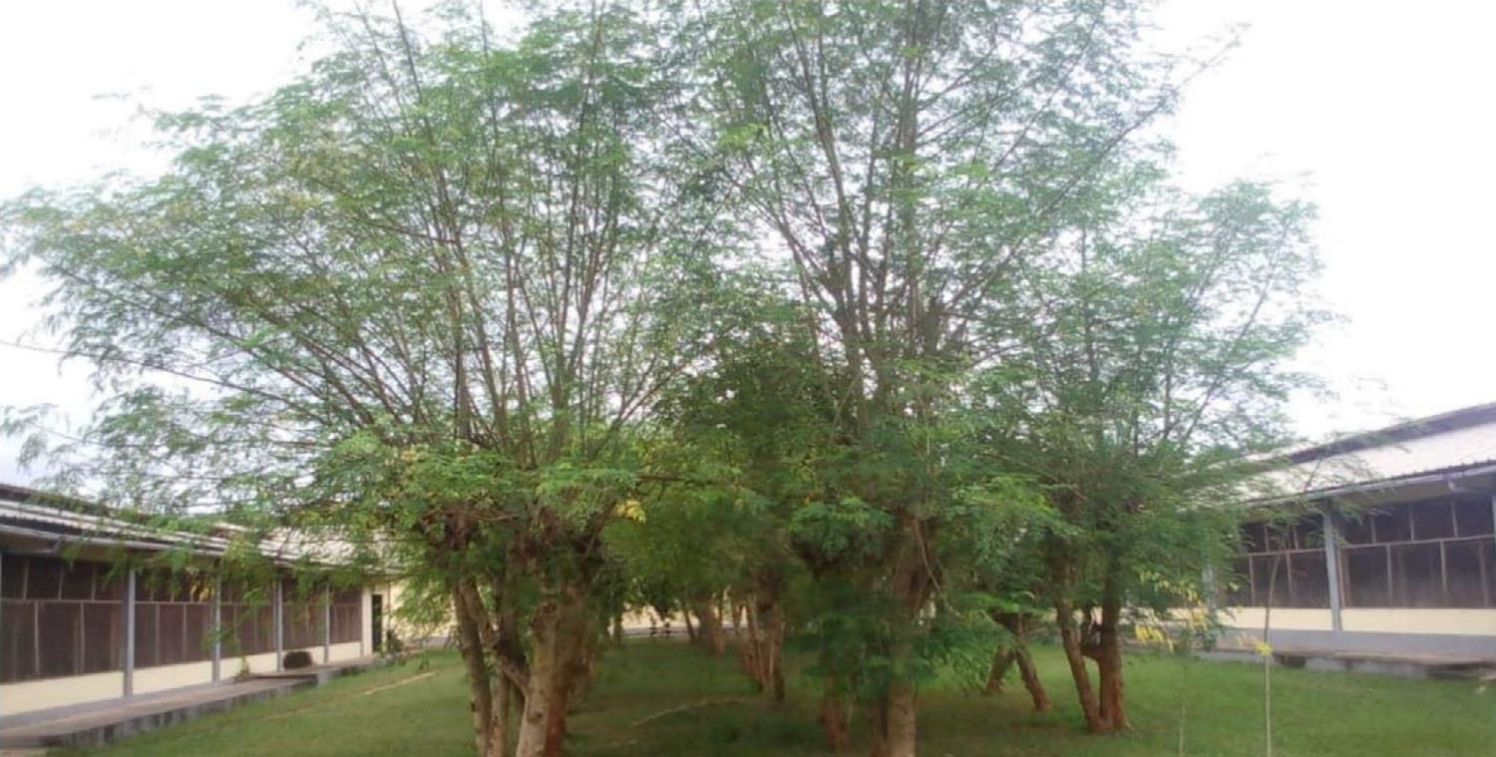
Tree shades behind poultry house for natural ventilation.

For poultry species like broiler birds that require less protection from climatic stressors, shade is a valuable technique as it blocks 30% of solar radiation (Rana et al., 2022) and substantially reduces heat load under heat stress conditions. Shades can improve animal comfort and productivity and should be designed to maximize ventilation and protection from the afternoon solar load (Nienaber & Hahn, 2007). As shown in Figure 5, natural ventilation generally refers to structures achieving airflow without fans; however, outdoor airflow is just as critical (Zhang et al., 2021). During cold weather, chill factors represent the combined effects of air temperature and air speed to represent greater convective heat loss from animals.

In hot weather, greater convective heat loss can be a matter of survival (Mader et al., 1999). Therefore, it is imperative that local features, such as windbreaks and even topography, be considered in pen design. A cornfield planted adjacent to a feedlot can block substantially more airflow than a short crop, such as alfalfa, when in the direction of the prevailing winds. The same can be said of locating pens on the leeward side of even a gentle slope.

### 5.2 Nutritional strategies

Sustainable feed sourcing and formulations can help minimize the environmental impact of broiler production (Ajayi et al., 2022; Oke et al., 2016; Oke et al., 2017; Oke et al., 2021b; Tallentire et al., 2018). Incorporating locally available feed ingredients, such as insect meal, algae and by‐products, can reduce the dependence on resource‐intensive feed ingredients and promote circular economy practices (Babu et al., 2022). Furthermore, precision feeding techniques and nutritional strategies can optimize feed conversion ratios and nutrient utilization, reducing feed wastage and environmental impacts (Ferket et al., 2002). Precision feeding involves tailoring the birds’ diet according to their specific growth stage and nutrient requirements, leading to improved feed efficiency and reduced environmental burdens (Ferket et al., 2002). Additionally, a nexus between improved gut health and the performance of chickens has been documented (El Sabry et al., 2023). Improvement in the gut could help alleviate the impact of climate change on birds.

Ensuring a constant and clean water supply is crucial for broilers to cope with thermal stress (Ahmad & Sarwar, 2006). Ample drinking water should be available at all times, as broilers tend to drink more in hot weather to regulate their body temperature. Regular maintenance and cleaning of waterers are necessary to prevent bacterial growth and ensure water quality. Adequate hydration supports the birds’ physiological functions and aids in heat dissipation, helping them cope with elevated temperatures. Additionally, phytogenic feed additives could be exploited for their antioxidant constituents to help mitigate the adverse impacts of heat stress (Akosile, Kehinde et al., 2023; Oke et al., 2017; Oke, 2018).

### 5.3 Genetic and epigenetic strategies

Breeding broilers for heat tolerance and disease resistance enhances their adaptability to changing climatic conditions (Hoffmann, 2010). Genetic selection programmes can focus on traits that improve the birds’ ability to thrive in hot environments (Cheng, 2010; Mirkena et al., 2010). Preserving genetic diversity within broiler species helps maintain resilience to environmental challenges. Protecting and utilizing genetic resources is essential for adapting broiler production to the changing climate. Major thermotolerant genes, including the naked neck, frizzle feather and dwarf genes of indigenous chickens in tropical environments, can be exploited for broiler chickens in these regions. The frizzle (F) and naked neck (Na) genes are thought to be candidates for temperature stress tolerance. They offer a practical, sustainable and cost‐effective solution to the heat stress challenge (Goel, 2021). Utilizing beneficial heat‐resistant genes such as frizzle (F) and naked neck (Na) might increase heat tolerance, growth performance and reproductive qualities in chickens (Fathi et al., 2022; Lin et al., 2006). Breeding for slower growth that will be more adapted to warmer temperatures could be beneficial. Moreover, to ensure sustainable livestock production in the face of climate change, research must focus on discovering numerous candidate genes like heat shock protein 70, which codes for thermotolerance (Abare et al., 2023; Balakrishnan et al., 2022). In addition to this, epigenetic strategies such as perinatal thermal manipulation could help in the thermotolerance of broiler chickens (Bilalissi et al., 2022; Meteyake et al., 2020; Oke et al., 2020; Oke et al., 2021b; Tona et al., 2022).

### 5.4 Managerial strategies

To alleviate thermal stress, broiler producers can employ various cooling methods. Evaporative cooling systems, such as misters, foggers or sprinklers, are commonly used to lower the temperature and increase humidity in the broiler house (Liang et al., 2020). These systems create a cooling effect as water evaporates, creating a more comfortable environment for the birds. Additionally, providing shaded areas within the house or using reflective roofing materials helps reduce heat absorption from sunlight, further mitigating heat stress (Khare et al., 2021).

Proper bedding and litter management play a vital role in heat management (Almeida Paz et al., 2010). Choosing appropriate bedding materials with good moisture‐absorbing properties helps reduce humidity levels in the broiler house (Kaukonen et al., 2017). This prevents excessive moisture build‐up, which can contribute to heat stress. Regularly removing and replacing soiled litter is essential to prevent the accumulation of heat‐trapping ammonia and maintain a dry and comfortable environment for the birds.

Adjusting management practices can significantly mitigate thermal stress in broiler production systems (Nienaber & Hahn, 2007). Scheduling feeding times during cooler periods of the day ensures that broilers consume their feed when temperatures are lower, reducing the heat generated during digestion (Daghir, 2008). Regular health checks allow early identification and intervention for any issues caused by heat stress, ensuring the well‐being of the birds (Madzingira, 2018). By creating a comfortable and controlled environment, broiler producers can optimize broiler performance, reduce the risk of heat‐related health issues and ensure the sustainability of the industry in the face of elevated temperatures. Continuous research and innovation in heat management strategies are vital for the long‐term success and resilience of broiler production in tropical climates.

## 6 POLICY IMPLICATIONS AND FUTURE DIRECTIONS IN CLIMATE CHANGE AND BROILER PRODUCTION

Broiler production needs to be addressed in a comprehensive way that takes into account policy frameworks, research and technology improvements. In the context of climate change, this study examines the policy ramifications and feasible actions to enhance sustainable broiler production. It also explores the industry's possibilities for the future in the tropics, suggesting potential areas for more study and innovation. Some policy frameworks and interventions that would promote sustainable broiler production in the tropics include:

### 6.1 Climate‐conscious agriculture policies

Governments can enact regulations that support climate‐conscious agricultural practices, such as the production of broilers. Incentives for implementing energy‐efficient housing systems, using renewable energy sources, and sustainable feed procurement are a few examples of these programmes. In addition, measures for carbon footprint might be implemented to encourage the industry's reduction in GHG emissions.

### 6.2 Environmental laws

To track and manage emissions from broiler‐producing sites, more environmental laws might be put in place. In order to reduce the negative environmental effects of broiler farming, these restrictions would promote the implementation of sustainable practices and technology.

### 6.3 Research and extension services

For broiler farmers to have access to the most recent information on climate change adaptation tactics, it is critical to invest in research and extension programmes. In order to spread information on heat stress management, nutritional interventions and disease prevention, governments should fund research institutes and extension initiatives. Some potential areas for further research and technological advancements include:

- Broiler strains that can flourish in tropical settings must be developed via research on breeding and genetic selection for heat tolerance. Finding genetic markers linked to heat tolerance would hasten the evolution of resilient broilers.
- Research into climate‐smart feed formulations can result in diets that are improved in terms of nutrient utilization and GHG emissions from feed production and support sustainability in the broiler sector.
- Intelligent monitoring and management technology. Technologies like remote monitoring systems and management tools powered by artificial intelligence can maximize broiler output and minimize resource waste. Real‐time monitoring of the environment, feed intake and health status is made possible by these technologies, enabling fast action when necessary.

## 7 CONCLUSION AND FUTURE PROSPECTS

The future of sustainable broiler manufacturing in the tropics is bright, despite the problems faced by climate change. The industry can overcome many of the present obstacles by adopting innovative technology and climate‐conscious practices. Increased production efficiency and a smaller carbon footprint may be achieved using heat‐tolerant broiler strains, improved feed formulas and cutting‐edge management systems. The industry's sustainability may also be improved by using circular economy ideas, such as employing insect‐based feed and recycling waste products.

Governments, academic institutions and the commercial sector are working together to bring about revolutionary improvements and advance sustainable broiler production. Establishing well‐planned strategies and interventions is required because climate change influences broiler output. For promoting sustainable broiler production, climate‐conscious agricultural policies, environmental laws and research funding are essential. The development of heat‐tolerant broilers and resource optimization both depend on advances in genetics and breeding, nutrition and technology. A resilient and sustainable broiler industry in the tropics will be possible if stakeholders seize these possibilities and collaborate more effectively. The future of broiler production may be guided towards increased productivity, less environmental impact and enhanced economic viability by proactively addressing climate change problems.

## AUTHOR CONTRIBUTIONS

All authors contributed to the study's conception and design. Material preparation, data collection and analysis were performed by Oyegunle Emmanuel Oke, Oluwaseun Ayomide Akosile, Victoria Anthony Uyanga, Folasade Olukemi Oke, Aderanti Ifeoluwa Oni, Kokou Tona and Okanlawon Mohammed Onagbesan. The first draft of the manuscript was written by Oyegunle Emmanuel Oke, Oluwaseun Ayomide Akosile, Victoria Anthony Uyanga, Folasade Olukemi Oke, Aderanti Ifeoluwa Oni, Kokou Tona and Okanlawon Mohammed Onagbesan, and all authors commented on previous versions of the manuscript. All authors read and approved the final manuscript.

## CONFLICT OF INTEREST STATEMENT

The authors have no relevant financial or non‐financial conflicts of interest to disclose.

## FUNDING INFORMATION

None.

### ETHICS STATEMENT

None.

### PEER REVIEW

The peer review history for this article is available at https://publons.com/publon/10.1002/vms3.1416.

## Data Availability

The data that support the findings of this study are available from the corresponding author upon reasonable request.
